# Detailed overview on the mutations detected by and the sensitivity of the GeneReader NGS sequencing platform

**DOI:** 10.1016/j.dib.2018.04.114

**Published:** 2018-05-04

**Authors:** Jessica Lüsebrink, Monika Pieper, Ramona-Liza Tillmann, Michael Brockmann, Oliver Schildgen, Verena Schildgen

**Affiliations:** Kliniken der Stadt Köln gGmbH, Klinikum der Privaten Universität Witten/Herdecke mit Sitz in Köln, Institut für Pathologie, Ostmerheimer Str. 200, D-51109 Köln/Cologne, Germany

## Abstract

This article presents additional next generation data from our pre-clinical validation study. In total 121 samples (clinical specimen and interlaboratory test samples) were tested successfully with next generation sequencing. 38 different mutations in six different genes were detected. Next to the detection of different mutations, the reproducibility of the NGS test was analyzed. Three samples were analyzed five times and the results were compared. Several mutations classified as non-pathogenic so far, have been detected repeatedly.

**Specifications table**

**Table:** 

Subject area	*Biology*
More specific subject area	*Genetics*
Type of data	*Tables*
How data was acquired	*Next Generation Sequencing*
Data format	*analyzed*
Experimental factors	*DNA was extracted from FFPE-tumor samples and interlaboratory test samples for further analysis*
Experimental features	*Next Generation Sequencing was performed to determine the presence of pathogenic mutations, which have an impact on therapeutic decisions*
Data source location	*Cologne, Germany*
Data accessibility	*with this article*

**Value of the data**

• **Common and rare mutations were detected, information on rare mutations could be of interest for further studies on specific mutations.**
• **Many mutations classified as non-pathogenic are detected alongside the analysis of pathogenic mutations, impact of those alterations on therapy is not fully known so far.**
• **Repeatability of the assay is given also for non-pathogenic mutations.**

## 1 Data

The data represented here is additional data from our pre-clinical validation study (1). Table 1 shows the mutations, which were detected by NGS in the samples used for a clinical pre-validation in our laboratory. In total 107 pathogenic mutations were detected in the 121 samples tested successfully. 38 different mutations were detected: 4 different mutations in BRAF, 12 in EGFR, 2 in KIT, 9 in KRAS, 5 in NRAS, and 6 in PIK3CA. Common mutations as KRAS c.35G>A (p.G12D), BRAF c.1799T>A (p.V600E) or EGFR c.2573T>G (p.L858R) could be detected as well as more uncommon mutations (e.g. KRAS c.351A>T (p.K117N) or NRAS c.437C>T (p.A146V)). Table 2 shows mutations, classified as non-pathogenic so far, which were detected in samples used for the testing of the repeatability of the GeneRead QIAact Actionable Insights Tumor Panel. 24 genetic alterations were detected in the Multiplex reference standard; nine respectively eight alterations were detected in the two clinical samples. Except for one mutation, which had a percentage of a mutant (PM) in the background of wild type in the range of the limit of detection, all mutations could be detected with similar frequencies in all five repetitions.

**Table 1:** Detected mutations. The table shows the mutations which were detected by NGS in the tested samples. Mutations were detected in BRAF, EGFR, KIT, KRAS, NRAS, and PIK3CA.

**Gene**	**Nucleotide substitution/ Amino Acid substitution**	**Total**	**%**	**Clinical specimen**	**Interlaboratory test samples**
**Total**		**107**	**100**	**44**	**63**
**BRAF**	**Total**	**18**	**16,82**	**7**	**11**
	c.1397G>C/ p.G466A	1	0,93	1	–
	c.1406G>C/ p.G469A	1	0,93	1	–
	c.1799T>A/ p.V600E	13	12,15	4	9
	c.1798_1799delGTinsAA/ p.V600K	3	2,80	1	2
**EGFR**	**Total**	**39**	**36,45**	**10**	**29**
	c.2127_2129delAAC/ p.E709_T710delinsD	1	0,93	–	1
	c.2156G>C/ p.G719A	1	0,93	1	–
	c.2254_227delTCTCC/ p.S725_I759del	1	0,93	1	–
	c.2222C>T/ p.P741L	1	0,93	1	–
	c.2237_2255delAATTAAGAGAAGCAACATCinsT/ p.E746_S752delinsV	3	2,80	1	2
	c.2235_2249delGGAATTAAGAGAAGC/ p.E746_A750del	3	2,80	3	–
	c.2248G>C/ p.A750P	1	0,93	–	1
	c.2281G>A/ p.D761N	1	0,93	–	1
	c.2369C>T/ p.T790M	12	11,21	1	11
	c.2573T>G/ p.L858R	11	10,28	–	11
	c.2582T>A/ p.L861Q	1	0,93	1	–
	c.2596G>A/ p.E866K	1	0,93	1	–
**KIT**	**Total**	**2**	**1,87**	**1**	**1**
	c.1509_1510insGCCTAT/ p.Y503_F504insAY	1	0,93	1	–
	c.2447A>T/p.D816V	1	0,93	–	1
**KRAS**	**Total**	**29**	**27,10**	**19**	**10**
	c.34G>A/ p.G12S	1	0,93	–	1
	c.34G>T/ p.G12C	2	1,87	2	–
	c.35G>A/ p.G12D	4	3,74	4	–
	c.35G>C/ p.G12A	2	1,87	1	1
	c.35G>T/ p.G12V	9	8,41	6	3
	c.38G>A/ p.G13D	4	3,74	2	2
	c.183A>T/ p.Q61H	4	3,74	2	2
	c.351A>T/ p.K117N	1	0,93	1	–
	c.436G>A/ p.A146T	2	1,87	1	1
**NRAS**	**Total**	**9**	**8,41**	**3**	**6**
	c.35G>A/ p.G12D	1	0,93	–	1
	c.181C>A/ p.Q61K	1	0,93	–	1
	c.182A>G/ p.Q61R	1	0,93	1	–
	c.182A>T/ p.Q61L	5	4,67	1	4
	c.437C>T/ p.A146V	1	0,93	1	–
**PIK3CA**	**Total**	**10**	**9,35**	**4**	**6**
	c.1633G>A/ p.E545K	5	4,67	2	3
	c.317_318delGCinsTT/ p.G106V	1	0,93	1	–
	c.331A>G/ p.K111E	1	0,93	1	–
	c.3140A>G/ p.H1047R	1	0,93	–	1
	c.3129G>T/ p.M1043I	1	0,93	–	1
	c.263G>A/ p.R88Q	1	0,93	–	1

**Table 2:** Non-pathogenic mutations detected with NGS in samples used to determine the precision. Several alterations not known to have therapeutically impact so far were detected with NGS.

**Sample**	**Mutation**		**Detected frequency of mutation [%]**					**Mean**	**SD**
			**Repetition 1**	**Repetition 2**	**Repetition 3**	**Repetition 4**	**Repetition 5**		
**Multiplex reference standard**	ALK	c.2535T>C/ p.G845G	82	80	84	84	83	82,60	1,50
	ALK	c.3036G>A/ p.T1012T	12	7,34	12	10	12	10,67	1,84
	ALK	c.4338C>T/ p.T1446T	33	31	33	30	34	32,20	1,47
	ALK	c.4472A>G/ p.K1491R	12	11	10	11	8,98	10,60	1,03
	BRAF	c.1929A>G/ p.G643G	18	19	21	21	20	19,80	1,17
	EGFR	c.1968C>T/ p.H656H	8,64	6,78	6,02	7	5,95	6,88	0,97
	EGFR	c.474C>T/ p.N158N	80	79	78	80	81	79,60	1,02
	EGFR	c.2361G>A/ p.Q787Q	14	16	16	16	15	15,40	0,80
	ERBB2	c.1963A>G/ p.I655V	37	33	33	34	33	34,00	1,55
	ERBB2	c.3508C>G/ p.P1170A	20	18	16	19	22	19,00	2,00
	ERBB3	c.3355A>T/ p.S1119C	34	34	30	30	33	32,20	1,83
	ESR1	c.1369+13777T>G	10	8,77	13	9	10	10,15	1,51
	ESR1	c.30T>C/ p.S10S	16	21	24	9	8,79	15,76	6,16
	KIT	c.2362-77G>A	32	35	30	31	29	31,40	2,06
	KIT	c.2586G>C/ p.L862L	8,36	8,7	8,99	9,23	9,3	8,92	0,35
	PDGFRA	c.1432T>C/ p.S478P	8,85	9,44	8,61	8,09	8,2	8,64	0,49
	PDGFRA	c.1701A>G/ p.P567P	100	100	100	100	100	100,00	0,00
	PDGFRA	c.1809G>A/ p.A603A	20	17	15	17	17	17,20	1,60
	PDGFRA	c.2472C>T/ p.V824V	18	15	14	15	20	16,40	2,24
	PDGFRA	c.612T>C/ p.N204N	9,2	8,68	10	8,91	8,34	9,03	0,56
	PDGFRA	c.939T>G/ p.G313G	8,71	7,99	8,88	8,64	10	8,84	0,65
	PIK3CA	c.-76-14537C>G	49	50	49	54	48	50,00	2,10
	PIK3CA	c.-77+8483C>T	6,72	7,54	7,09	7,5	7,3	7,23	0,30
	PIK3CA	c.1173A>G/ p.I391M	10	6,49	8,08	11	10	9,11	1,62
**Tumor sample 1**	KRAS	c.*2505T>G	47	47	46	50	51	48,20	1,94
	ALK	c.2535T>C/ p.G845G	44	43	43	44	44	43,60	0,49
	ALK	c.4472A>G/ p.K1491R	58	56	57	55	58	56,80	1,17
	EGFR	c.2361G>A/ p.Q787Q	47	51	52	48	48	49,20	1,94
	EGFR	c.474C>T/ p.N158N	50	49	51	53	50	50,60	1,36
	EGFR	c.2184+19G>A	4,96	–	5,5	–	–	5,23	0,27
	EGFR	c.1881-781C>T	49	54	50	49	50	50,40	1,85
	ERBB2	c.3508C>G/ p.P1170A	51	52	46	53	48	50,00	2,61
	PDGFRA	c.1701A>G/ p.P567P	100	100	99	98	99	99,20	0,75
**Tumor sample 2**	ALK	c.2535T>C/ p.G845G	47	47	45	41	43	44,60	2,33
	ALK	c.4472A>G/ p.K1491R	43	46	43	47	46	45,00	1,67
	EGFR	c.474C>T/ p.N158N	41	40	45	44	42	42,40	1,85
	EGFR	c.2361G>A/ p.Q787Q	99	99	98	99	99	98,80	0,40
	ERBB2	c.3508C>G/ p.P1170A	47	47	46	51	46	47,40	1,85
	ESR1	c.30T>C/ p.S10S	99	100	100	100	100	99,80	0,40
	KIT	c.2362-77G>A	34	33	36	34	37	34,80	1,47
	PDGFRA	c.1701A>G/ p.P567P	100	100	100	100	100	100,00	0,00

## 2 Experimental design, materials and methods

122 samples were tested retrospectively to validate the use of the GeneReader System in our laboratory. One sample proofed to be not suitable for NGS testing and was excluded from the analysis. The GeneRead QIAact Actionable Insights Tumor Panel was used for target enrichment of ALK, BRAF, EGFR, ERBB2, ERBB3, ESR1, KIT, KRAS, NRAS, PDGFRA, PIK3CA, and RAF1. Following target enrichment, library preparation and clonal amplification was done using the GeneRead DNA Library Q Kit and the GeneRead Clonal Amp Q Kit. DNA quality and concentration was determined after target enrichment and library preparation with the QIAxcel DNA High Resolution Kit on the QIAxcel instrument. Sequencing was performed on the GeneReader instrument with the GeneRead Sequencing Q Kit. Qiagen Clinical Insight Software was used for analysis and interpretation of the results. Sequencing products are analyzed by comparison with the following reference sequences: NM_004304.4 (ALK), NM_004333.4 (BRAF), NM_005228.4 (EGFR), NM_004448.3 (ERBB2), NM_001982.3 (ERBB3), NM_001122742.1 (ESR1), NM_000222.2 (KIT), NM_004985.4 (KRAS), NM_002524.4 (NRAS), NM_006206.5 (PDGFRA), NM_006218.3 (PIK3CA), and NM_002880.3 (RAF1).
