# Supplementary material for: Detailed overview on the mutations detected by and the sensitivity of the GeneReader NGS sequencing platform
Source: Data Brief. 2018 May 4;18:1962–6. doi: 10.1016/j.dib.2018.04.114 (PMC5998651; doi:10.1016/j.dib.2018.04.114)
Supplement: Supplementary file 1 — Supplementary material [file mmc1.doc]

None of the authors has any conflict of interest. Unfortunately the COI form was not downloadable (missing link), please send us the for if required during the production process, we will immediately take care for it.
